# A two-stage approach to the depot shunting driver assignment problem with workload balance considerations

**DOI:** 10.1371/journal.pone.0181165

**Published:** 2017-07-13

**Authors:** Jiaxi Wang, Manfred Gronalt, Yan Sun

**Affiliations:** 1 School of Traffic and Transportation, Beijing Jiaotong University, Beijing, People's Republic of China; 2 Institute of Production and Logistics, University of Natural Resources and Life Sciences, Vienna (BOKU), Vienna, Republic of Austria; 3 School of Management Science and Engineering, Shandong University of Finance and Economics, Jinan, Shandong, People's Republic of China; Montclair State University, UNITED STATES

## Abstract

Due to its environmentally sustainable and energy-saving characteristics, railway transportation nowadays plays a fundamental role in delivering passengers and goods. Emerged in the area of transportation planning, the crew (workforce) sizing problem and the crew scheduling problem have been attached great importance by the railway industry and the scientific community. In this paper, we aim to solve the two problems by proposing a novel two-stage optimization approach in the context of the electric multiple units (EMU) depot shunting driver assignment problem. Given a predefined depot shunting schedule, the first stage of the approach focuses on determining an optimal size of shunting drivers. While the second stage is formulated as a bi-objective optimization model, in which we comprehensively consider the objectives of minimizing the total walking distance and maximizing the workload balance. Then we combine the normalized normal constraint method with a modified Pareto filter algorithm to obtain Pareto solutions for the bi-objective optimization problem. Furthermore, we conduct a series of numerical experiments to demonstrate the proposed approach. Based on the computational results, the regression analysis yield a driver size predictor and the sensitivity analysis give some interesting insights that are useful for decision makers.

## 1. Introduction

Nowadays, high-speed railways (HSR) play an important role in public transportation systems, mainly due to its environmentally friendly, energy-saving, comfortable, fast and safe characteristics. Therefore, many countries spare no effort to build new HSR lines. China, for example, has built an advanced HSR network with a scale of more than 20,000 km, making it the world's largest HSR network. Along with the continuous construction of HSR, the number of high-speed trains or electric multiple units (EMU) that are put into service increases rapidly, which remains a challenging task for HSR operators to design effective and efficient operation plans, such as rolling stocks circulation plans and train timetables. Furthermore, since maintenance activities are a critical point to ensure safe operations of EMU trains, the massive number of EMUs also introduces great difficulties to depot staff to accomplish maintenance task efficiently. Here EMU depots refer to workshops where high-speed trains get maintained. Currently, the maintenance schedules in EMU depots are mainly manually designed by depot dispatchers. As a result, it is a time-consuming and laborious work to obtain a feasible schedule, let alone an optimal one. In practice, the schedule making process always requires several hours of painstaking effort by a team of highly experienced dispatchers. Therefore, it is of great significance to generate maintenance schedules through automatic computing.

The goal of maintenance schedules is to guide the depot maintenance crew to execute maintenance work in a well-ordered way. Generally, these maintenance schedules can be classified into two categories: depot train routing plans and depot workforce scheduling plans. The first category focuses on the train movements within a depot, e.g., how to assign depot tracks to EMU trains, and how to shunt trains between tracks in a conflict-free manner, which is embodied in a shunting schedule [1] in most cases. The second category, clearly, aims at planning the depot workforce, such as shift scheduling and task assignment. Basically, the depot train routing plans and workforce schedules are correlated to some extent. However, since both planning problems belong to the NP-hard class, the train routing planning problem and the workforce scheduling problem are always solved sequentially. In this study, we explore the depot shunting driver assignment (DSDA) problem under the condition that a depot shunting schedule is given in advance. As for the DSDA problem, two aspects are discussed, namely, the workforce size of shunting drivers and the task assignment for shunting drivers. In order to link these two aspects, we propose a novel two-stage optimization framework.

**Fig 1:**
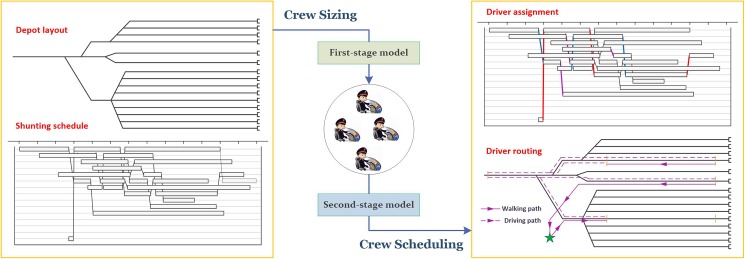
The two-stage optimization framework.

The overall optimization framework for the DSDA problem is illustrated by Fig 1. As depicted in the figure, the inputs of the optimization framework are primarily the depot infrastructure layout and depot shunting schedule; and the outputs include the driver assignment plan and the driver routing plan. Furthermore, the driver size serves as the intermediary that links the first stage and second stage; that is, the driver size is not only the output of the first stage but also the input of the second stage. Indeed, the proposed two-stage approach consists of two optimization models. The first-stage model aims to determine an optimal workforce size of shunting drivers, while the second-stage model is used to design the "best" assignment plan of shunting tasks for the drivers whose total number is computed in the first stage. The second-stage workforce scheduling subproblem is formulated as a bi-objective optimization model, in which we comprehensively consider the objectives of minimizing the total walking distance and maximizing the workload balance. The advantage of the bi-objective formulation is that depot decision makers can easily select a desirable or reasonable configuration from the solution pool when making tradeoffs between overall costs (total walking distance) and individual fairness (workload balance). Both models consider the depot track layout, walking distance and walking time of drivers, etc. Using the state-of-the-art optimization solver Gurobi, the solution times of the models are just a matter of seconds. Based on the computational results, we further reveal some interesting insights that can be useful for practitioners and operators of EMU depots, including a driver size predictor that is applicable for roughly determining a workforce size of shunting drivers while just using three easily obtainable problem characteristics, and a sensitivity analysis result that reports how the number of shunting tasks impacts the total walking distance as well as the workload balance.

The remainder of this paper is organized as follows. In Section 2, a literature review for recent studies on crew sizing and crew scheduling related problems is presented. Section 3 proposes a two-stage approach for the DSDA problem which aims to determine an optimal driver size as well as an optimal shunting task assignment plan. In Section 4, in order to generate Pareto solutions to the bi-objective model of the second stage, we introduce the normalized normal constraint method, and optimize it with a modified Pareto filter algorithm. Section 5 conducts a series of computational experiments and reveals some interesting insights based on the computational results. Finally, conclusions and research prospects are presented in Section 6.

## 2. Literature review

The DSDA problem can be regarded as a post-phase of the depot shunting scheduling problem. Although there is a lot of attention paid to the train shunting planning problem (see, e.g., [2–4]), literature on the shunting driver scheduling problem is scarce. Nevertheless, relevant studies on crew sizing problems and crew scheduling problems within the transportation planning field are worth referring to. Rich literature have discussed these two attractive topics, which lays a solid foundation on which this study is carried out. A comprehensive literature review is proceeded as follows to help the readers better understand the progress of related work.

### 2.1. Crew (workforce) sizing problem

The transportation crew sizing problem, which is also known as the workforce or personnel sizing problem, refers to determining an optimal crew size that is able to cover a given set of transportation tasks (e.g., train services, maintenance tasks) within a given time framework. This problem falls into the scope of human resource management [5]. Verbeek [6] proposed a mixed-integer mathematical formulation for the crew sizing problem arising in the strategic manpower planning of airline pilots. Shiftan and Wilson [7] presented a two-stage method for determining the optimal number of public transport operating personnel so as to minimize total operating costs subject to a constraint on the minimum level of service reliability. The first stage of the method estimated the optimal number of operators required in each period, and the second stage defined the annual hiring program and vacation allocation plan. Zak [8] presented the application of the methodology of multiple criteria decision making/aiding for the problem that optimizes the crew size in the mass transit system operated by a public transportation company. A multi-objective mathematical programing was formulated and then a customized heuristic procedure was then designed to solve the problem.

If we define the transportation mobile equipment (e.g., vehicles, rolling stocks and locomotives) as the “generalized crew”, the fleet sizing problem is similar with the crew sizing problem to some extent. The fleet sizing problem is also one of highlighted topics within the transportation area (see [9–11]). Taking the railway transportation as an example, the concept of fleet sizing embodies (but is not limited to) the following problems: the locomotive scheduling problem [12–15], the rolling stock circulation problem [16–18] and the empty car distribution problem [19–21]. Aside from the transportation planning field, the workforce sizing optimization has also been widely applied to other fields in recent years, to name but a few, the manufacturing industry field [22] and the nurse staffing field [23].

### 2.2. Crew scheduling problem

The crew scheduling problem refers to assigning *K* crew members to *N* tasks with fixed start and finish times such that each crew does not exceed a limit on the total time it can spend working [24]. Majority of the relevant studies assume that the operation staff is given in advance, i.e., the crew size *K* serves as a known parameter of the crew scheduling module. The crew scheduling problem occurs frequently in airline, railway and urban public transportation systems, which is commonly modeled as set covering problems. Because of the NP-hard complexity nature of this problem, many researchers have paid their attention to finding fast solution algorithms. Haase et al. [25] presented a branch-and-price solution approach (only for the crew schedules) for solving the simultaneous vehicle and crew scheduling problem in urban mass transit systems. Freling et al. [26] designed a flexible branch-and-price algorithm to address the problem of crew scheduling and crew rostering. Huisman et al. [27] presented two algorithms both of which were based on a combination of column generation and Lagrangian relaxation for integrated vehicle and crew scheduling in the multiple-depot case. Jütte and Thonemann [28] presented a column generation based decomposition algorithm, namely, the divide-and-price algorithm, which achieved high-quality solutions at reasonable runtimes for the railway crew scheduling problem. In addition, (meta-) heuristics are also widely adopted to solve the crew scheduling problem, which have showed their great potential on the aspect of computational efficiency compared with exact algorithms. Deng and Lin [29] introduced an ant colony optimization algorithm to solve the airline crew scheduling problem. Performance was evaluated by performing computational tests regarding real cases as the test problems. Azadeh et al. [30] presented a particle swarm optimization (PSO) algorithm synchronized with a local search heuristic for solving the airline crew scheduling problem. Computational results showed the effectiveness and superiority of the proposed hybrid PSO algorithm over other algorithms (e.g., genetic algorithm). Hanafi and Kozan [31] applied a hybrid constructive heuristic with the simulated annealing search algorithm to the railway crew scheduling problem. The performance of the algorithms was evaluated by applying computational experiments on randomly generated test instances.

In this paper, we aim to simultaneously consider the crew sizing problem and the crew scheduling problem within a two-stage modeling framework. Furthermore, on the crew scheduling stage, we propose a novel bi-objective optimization model to comprehensively take into account the objectives of overall costs (measured by total walking distance) and individual fairness (indicated by workload balance). To the best of our knowledge, few previous studies have carefully addressed the DSDA problem that involves both the workforce sizing module and the crew scheduling module while comprehensively considering the overall cost and the workload balance objectives. Another contribution of this paper is that we apply the normalized normal constraint method combined with a modified Pareto filter scheme to generate Pareto solutions to the bi-objective optimization problem. Last but not least, our discussions with respect to the computational results of a set of problem instances provide a shunting driver size predictor and some sensitivity analysis results that are useful for decision makers when designing shunting driver assignment plans.

## 3. Model formulation

In this section, we shall first give a brief introduction to the maintenance activities in the high-speed railway system. A formal definition of the depot shunting driver assignment problem is then given. After that, we present mathematical formulations for the problem within a two-stage optimization framework. The computational complexity of both models are also discussed in this section.

### 3.1. Problem description and notations

**Fig 2:**
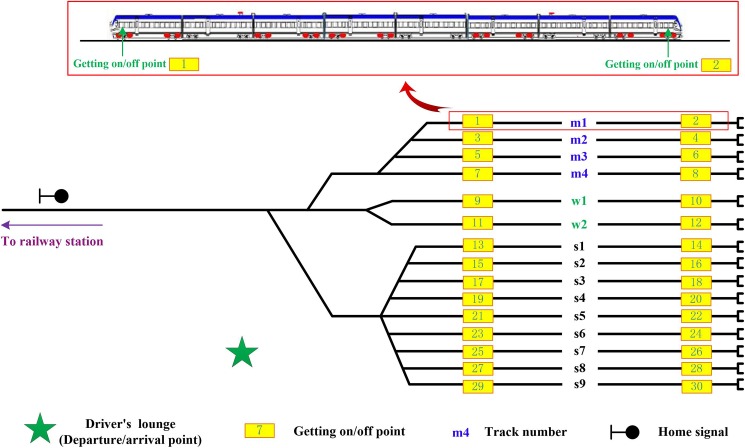
A typical depot track layout with getting on/off points.

Maintenance activities play a critical role in railway transportation to ensure safe operations. There exist various types or levels of maintenance work in repair workshops, which usually follows a time-cycle or a distance-cycle manner. For example, in China high-speed railway system, the maintenance work for EMU trains is divided into five levels. Each level of maintenance corresponds to a time cycle as well as a distance cycle. For instance, the time cycle and the distance cycle of the first-level maintenance are two days and 3,000 km, which means when a train’s accumulative operation time from last maintenance reaches two days or its accumulative running distance reaches 3,000 km, the train needs to complete the first-level maintenance. The first- and second-level are called the minor maintenance while the remainder are defined as the major maintenance. The minor and major maintenance work are carried out in minor maintenance depots and major maintenance workshops, respectively. In this paper, we focus our attention on the minor maintenance work. One of the most important plans to guide depot staff to perform the maintenance work is the so-called depot shunting schedule. For a detailed description of the depot shunting schedule, we refer the readers to a previous paper [1]. After generating a shunting schedule, maintenance tasks are then assigned. There are different worker groups that are responsible for different types of maintenance related work, e.g., the train inspection and repair group, the train cleaning group, and the train shunting group. The train shunting group refers to a fleet of shunting drivers that are in charge of train shunting tasks. A detailed shunting task involves two fundamental aspects: time windows of the task (time information) and locations to perform the task (space information). The time windows can be derived from the shunting schedule directly; and with considerations of the depot track layout (see Fig 2), the locations can be also determined. To have a better understanding of the relationship between a depot shunting schedule and a depot shunting driver assignment plan, we use the following illustrations to give more details.

**Fig 3:**
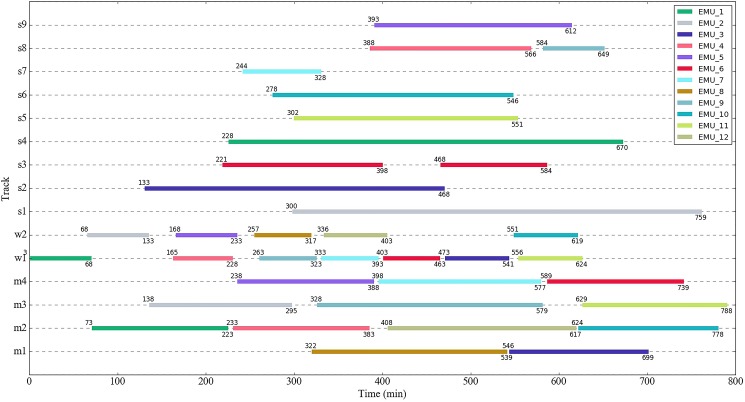
Diagram of the depot shunting schedule.

Fig 3 depicts a shunting schedule corresponding to the depot track layout shown in Fig 2. In this shunting schedule, the connection between a dwell on one track and a subsequent dwell on another track implies a shunting movement. For example, at the time moment 68, EMU_1 (colored with dark green) starts a shunting movement from track w1; then it is shunted towards track m2. At the time moment 73 the train finishes the shunting movement and stays on track m2. In this way, the shunting schedule tells the exact time stamps (time information) of a shunting task.

**Fig 4:**
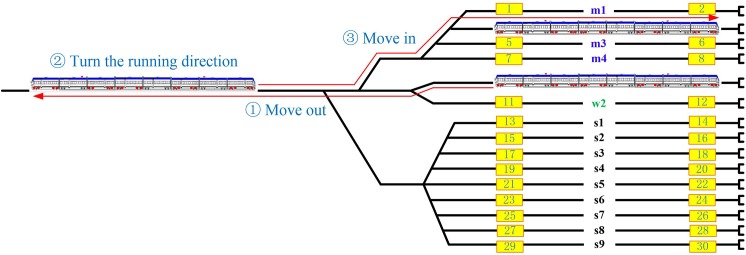
A shunting movement from track w1 to track m2.

We then turn our attention to how we can get the space information of a shunting task. It is known that an EMU train is able to move towards both directions using its own engine. Therefore, a train always has two driver cabs, located at the head of the train and the tail of the train (see Fig 2). The shunting driver who controls the train needs to be in the cab corresponding to the train’s running direction. If a train moves forward, the driver uses the “head cab”; and vice versa. Since a shunting movement (see Fig 4) always needs to turn the running direction of a train, the shunting driver will use both cabs. As a result, the getting on point where a driver starts a shunting movement is usually not identical to the getting off point where a shunting movement finishes. For a dead-end type of depot layout (see Fig 2), the getting on points (numbered with odd numbers in Fig 2) are near the bottleneck of the depot while the getting off points (numbered with even numbers in Fig 2) are near the ends of the tracks. By mapping the shunting schedule to the track layout, we can obtain the space information of a shunting task. For example, the above-mentioned shunting movement from track w1 to track m2 of EMU_1 implies that the shunting driver’s getting on point is 9 while the getting off point is 4. Up to this point, we have obtained both the time and space information for a shunting task.

One shunting driver is capable of handling several shunting tasks. In order to perform another shunting task after completing one, the shunting driver has to walk from the current getting off point to the corresponding getting on point for the next shunting task within a given time window. Before starting and after finishing all assigned shunting tasks, the driver also needs to walk from and to the driver's lounge. Note that, at the direction turning step (step ② in Fig 4) during a shunting process, the driver needs to get off from the head driver cab and walk to the tail cab, and then get on to the tail cab and the tail cab therefore becomes the head cab. Since a shunting movement is only assigned to one driver, the walking effort of the driver during the train direction turning process is mandatory. For simplification reasons, in the optimization model presented in next section, we will treat the shunting movement as a black box and merge the driver's walking effort into the driver's driving effort. The shunting driver assignment plan aims to design an optimal partition of the set of shunting tasks and optimal connections of shunting tasks for each resulting subset while satisfying various operational requirements.

Before we present the mathematical model to describe the shunting driver assignment problem, we introduce the following notations used in our formulations. Let *P* be the set of driver's getting on/off points indexed by *p* and *q*, *P* = {0, 1, 2, …, *m*}, where *m* is the total number of getting on/off points. Kindly note that we assume all shunting drivers’ departure and arrival points are identical, i.e., the driver's lounge denoted by a five-pointed star in Fig 2. We use element “0” in set *W* to represent the point. Let *S* be the set of shunting tasks indexed by *i* and *j*, *S* = {0, 1, 2, …, *n*}, where *n* is the total number of shunting tasks. For the sake of modeling convenience, here we add the element of “0” to set *S* to represent the virtual task that links the arrival and departure activities of the shunting drivers at the driver lounge. Let *W* denote the set of shunting drivers indexed by *k*, *W* = {1, 2, …, *c*}, where *c* is the workforce size of shunting drivers. We define the start point (getting on point) and end point (getting off point) of shunting task *i* as *sp*_*i*_ and *ep*_*i*_, respectively. The walking distance between point *p* and *q* is denoted by *d*(*p*,*q*). We assume that the walking speed of all shunting drivers is identically *v*. Meanwhile, we define the start time and end time of shunting task *i* as *st*_*i*_ and *et*_*i*_, respectively. We use binary decision variables *x*_*ij*_ to decide whether shunting tasks *i* and *j* are connected, where *x*_*ij*_ = 1 if *i* and *j* are connected, *x*_*ij*_ = 0 otherwise. Binary decision variables $y_{ij}^{k}$ are used to describe whether shunting tasks *i* and *j* are connected by shunting driver *k*; if *i* and *j* are connected by *k*, it takes value one, otherwise it takes value zero. Furthermore, we use auxiliary binary decision variables *z*_*ik*_ to model whether shunting task *i* is assigned to shunting driver *k*; when *i* is assigned to *k*, its value is one, otherwise its value is zero.

### 3.2. The First-stage model

In the first-stage of the DSDA problem, we aim to find an optimal workforce size of shunting drivers to complete all the shunting tasks within a predefined shunting schedule. The fist-stage crew sizing sub-problem is formulated as an Integer Linear Programming (ILP) model. The objective function and constraints of the model can be written as follows:

*First-stage Model* *(FSM)*

- ***Objective function***

Minimizing the workforce size of shunting drivers:

minc (1)

- ***Subject to***

Each shunting task should be assigned to one shunting driver:

$$\sum_{i\in S}x_{ij}=1\;\forall j\in S\backslash \left\{0\right\}$$ (2)

$$\sum_{j\in S}x_{ij}=1\;\forall i\in S\backslash \left\{0\right\}$$ (3)

All shunting drivers’ departure and arrival points are identically the driver's lounge:

$$\sum_{i\in S}x_{i0}=c$$ (4)

$$\sum_{j\in S}x_{0j}=c$$ (5)

The walking time from the getting off point of a shunting task to the getting on point of the connected shunting task should be less than the time interval of the two tasks; otherwise, the two shunting tasks should never be connected, i.e., *x*_*ij*_ = 0:

$$\left(st_{j}-et_{j}-\frac{d(ep_{i},sp_{j})}{v}\right)\cdot x_{ij}\geq 0\;\forall i,j\in S\backslash \left\{0\right\}$$ (6)

Decision variables are binary:

$$x_{ij}\in \left\{0,1\right\}\;\forall i,j\in S$$ (7)

The computational complexity of the first-stage model (FSM) can be analyzed as follows. Generally, two aspects are discussed with respect to the model complexity: decision variables and constraints. Clearly, the complexity of the decision variables is *O*(*n*^2^). In contrast, the complexity of constraints (2) and (3) is *O*(*n*) while the complexity is *O*(*n*^2^) for constraint (6).

### 3.3. The Second-stage model

In the second-stage model, we aim to minimize the total walking distance as well as to balance the workload for the shunting drivers whose total number *c* is computed in the first-stage model. We propose a bi-objective modeling framework to consider both goals. By removing auxiliary decision variables, the basic mathematical model can be modified to a more concise formulation without increasing the computational complexity.

#### 3.3.1. Bi-objective formulation

We formulate the second-stage problem as a Bi-objective Binary Integer Quadratic Programming (BBIQP) with two groups of decision variables. The objective functions and constraints can be written as follows:

*Second-stage Model 1* *(SSM_1)*

- ***Objective function***

Objective 1: Minimizing the total walking distance of all shunting drivers

$$\min\;Z_{1}=\sum_{i\in S}\sum_{j\in S}\sum_{k\in W}d(ep_{i},sp_{j})\cdot y_{ij}^{k}$$ (8)

Objective 2: Minimizing the imbalance of workload for the shunting drivers

Here, we define the actual workload of a shunting driver as the sum of driving time and walking time. Let *aw*_*k*_ denote the actual workload of driver *k* and it is calculated by the following equation:

$$aw_{k}=\sum_{i\in S\backslash \left\{0\right\}}(et_{i}-st_{i})\cdot z_{ik}+\sum_{i\in S}\sum_{j\in S}\frac{d(ep_{i},sp_{j})}{v}\cdot y_{ij}^{k}$$ (9)

One advantage of this definition is that it benefits from considering the redundant walking time, which equals the time interval of two shunting tasks minus the actual walking time. Under such a definition, we can now turn our attention to the objective formulation. It is a widely used method to measure the imbalance with a variance metric. Accordingly, we formulate the second objective, namely, minimizing the imbalance of actual workload for the shunting drivers as follows:

$$\min\;Z_{2}=\sum_{k\in W}{\left(aw_{k}-\frac{1}{c}\sum_{k\in W}aw_{k}\right)}^{2}$$ (10)

According to the original definition of variance, the right side of formula (10) should be divided by the scale of data samples (in this model the scale is *c*). The scale is a constant, which means that it has no effect on the optimal solution (task assignment plan). For simplification reasons, we remove the constant from formula (10).

- ***Subject to***

Each shunting task should be assigned to one shunting driver:

$$\sum_{k\in W}z_{ik}=1\;\forall j\in S\backslash \left\{0\right\}$$ (11)

All shunting drivers’ departure and arrival points are identically the driver's lounge:

$$\sum_{k\in W}z_{0k}=c$$ (12)

The walking time between two connected shunting tasks should be less than the time interval of the tasks:

$$\left(st_{j}-et_{i}-\frac{d(ep_{i},sp_{j})}{v}\right)\cdot y_{ij}^{k}\geq 0\;\forall i,j\in S\backslash \left\{0\right\},k\in W$$ (13)

The two groups of decision variables have the following relationship:

$$\sum_{j\in S}y_{ij}^{k}=z_{ik}\;\forall i\in S,k\in W$$ (14)

$$\sum_{i\in S}y_{ij}^{k}=z_{jk}\;\forall j\in S,k\in W$$ (15)

Decision variables are binary:

$$y_{ij}^{k}\in \left\{0,1\right\}\;\forall i,j\in S,k\in W$$ (16)

$$z_{ik}\in \left\{0,1\right\}\;\forall i\in S,k\in W$$ (17)

Similarly, the computational complexity of the model (SSM_1) is analyzed as follows. The complexity of constraints (11), (13), (14) and (15) is *O*(*n*), *O*(*cn*^2^), *O*(*cn*) and *O*(*cn*), respectively. It is observed that the complexity of decision variables $y_{ij}^{k}$ is *O*(*cn*^2^) while the complexity of auxiliary decision variables *z*_*ik*_ is *O*(*cn*).

#### 3.3.2. Model reformulation without auxiliary decision variables

In fact, the SSM_1 can be improved to a more concise formulation without using auxiliary decision variables *z*_*ik*_. We define the resulting reformulation as Second-stage Model 2 (SSM_2). The detailed mathematical formulations of SSM_2 is presented as follows:

*Second-stage Model 2* *(SSM_2)*

- ***Objective function***

Objective 1:

Formula (8)

Objective 2:

Formula (10)

where *aw*_*k*_ is rewritten as follows:

$$aw_{k}=\sum_{i\in S\backslash \left\{0\right\}}\left((et_{i}-st_{i})\cdot \sum_{j\in S}y_{ij}^{k}\right)+\sum_{i\in S}\sum_{j\in S}\frac{d(ep_{i},sp_{j})}{v}\cdot y_{ij}^{k}$$ (18)

- ***Subject to***

Each shunting task should be assigned to one shunting driver:

$$\sum_{i\in S}\sum_{k\in W}y_{ij}^{k}=1\;\forall j\in S\backslash \left\{0\right\}$$ (19)

$$\sum_{j\in S}\sum_{k\in W}y_{ij}^{k}=1\;\forall i\in S\backslash \left\{0\right\}$$ (20)

$$\sum_{i\in S}y_{ij}^{k}-\sum_{i\in S}y_{ji}^{k}=0\;\forall j\in S,k\in W$$ (21)

All shunting drivers’ departure and arrival points are identically the driver's lounge:

$$\sum_{i\in S}y_{i0}^{k}=1\;\forall k\in W$$ (22)

$$\sum_{j\in S}y_{0j}^{k}=1\;\forall k\in W$$ (23)

The walking time should be less than the time interval of two connected shunting tasks:

$$\left(st_{j}-et_{i}-\frac{d(ep_{i},sp_{j})}{v}\right)\cdot y_{ij}^{k}\geq 0\;\forall i,j\in S\backslash \left\{0\right\},k\in W$$ (24)

Decision variables are binary:

$$y_{ij}^{k}\in \left\{0,1\right\}\;\forall i,j\in S,k\in W$$ (25)

The complexity of constraints (19) ~ (24) is *O*(*n*), *O*(*n*), *O*(*c*), *O*(*c*), *O*(*n*) and *O*(*cn*^2^), respectively. Clearly, the complexity of decision variables is *O*(*cn*^2^). Compared with SSM_1, the complexity of constraints and decision variables has not increased. However, the total number of decision variables has been significantly reduced, which makes the problem easier to solve. In our paper, we shall use SSM_2 to address the second-stage sub-problem, i.e., the shunting driver routing and scheduling problem.

## 4. Pareto optimality for the bi-objective optimization problem

The second-stage model proposed in Section 3 addresses a bi-objective optimization problem (BOP). In most cases, the two objectives of BOPs are conflicting, which requires the decision makers to make a tradeoff between them [32]. There are various approaches available in scientific literature (see, for example, [33]) to help decision makers make reasonable tradeoffs (i.e., obtain solutions), e.g., sequential optimization method, weighting method and goal programming method. Using different solution approaches, decision makers are able to obtain different configurations from the BOP solution pool. The generally accepted solution of a BOP is said to be Pareto optimal, or a Pareto solution. A Pareto solution is one for which any improvement in one objective can only take place if at least one other objective worsens [34]. By generating a set of Pareto solutions, one can obtain the so-called Pareto frontier. In this way, it is quite easy for decision makers to get suitable solutions from the Pareto frontier. Therefore, we will focus on generating the Pareto frontier for the second-stage model. In general, Pareto frontier generation methods can be categorized into two groups: classical methods and evolutionary methods. For a recent overview of Pareto frontier generation methods, we refer the readers to the corresponding section of reference [35]. In this study, we use the normalized normal constraint method proposed by Messac et al. [34] to generate the Pareto frontier for the second-stage BOP (i.e., SSM_2). The normalized normal constraint method includes two phases: Pareto points generation and non-Pareto solutions filter. In the following, we first give a brief introduction to the Pareto points generation phase, and then develop a modified Pareto filter algorithm in the second phase.

### 4.1. Pareto points generation

Given the bi-objective optimization problem composed of formulae (8), (10) and (18) ~ (25), the normalized normal constraint method for generating Pareto points is described as follows.

**Fig 5:**
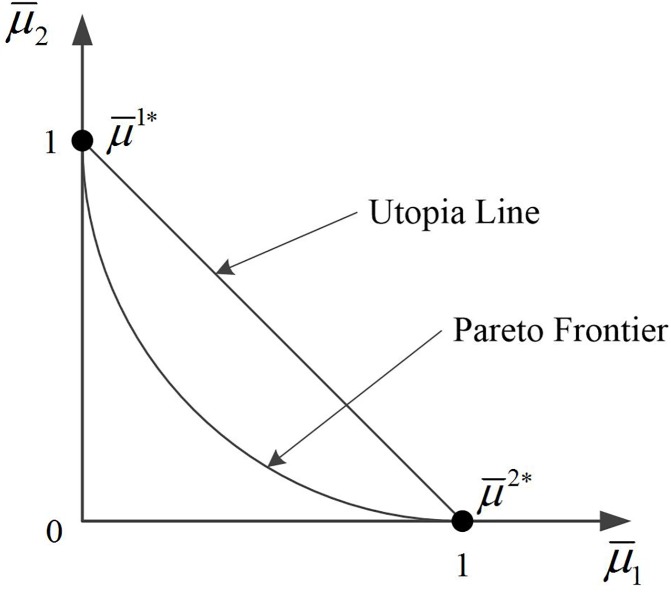
The Pareto frontier and the Utopia line in the normalized objective space.

First, by solving the two single-objective optimization problems (one problem is composed of formulae (8) and (18) ~ (25); and another one is composed of formulae (10) and (18) ~ (25)) separately, we can gain their respective optimal solutions *x*^1*^ and *x*^2*^. Let *μ*_1_ and *μ*_2_ represent Objective 1 (formula (8)) and Objective 2 (formula (10)), respectively. To avoid scaling deficiencies, the objective functions *μ*_1_ and *μ*_2_ should be normalized by the following equation:

$$\bar{\mu }=\left\{\frac{\mu_{1}(x)-\mu_{1}(x^{1*})}{\mu_{1}(x^{2*})-\mu_{1}(x^{1*})}\;\frac{\mu_{2}(x)-\mu_{2}(x^{2*})}{\mu_{2}(x^{1*})-\mu_{2}(x^{2*})}\right\}$$ (26)

where $\bar{\mu }$ is called the normalized design metrics (objective functions). If we substitute the optimal solutions *x*^1*^ and *x*^2*^ into Eq (26), we obtain two points: ${\bar{\mu }}^{1*}=\bar{\mu }(x^{1*})=[0,1]$ and ${\bar{\mu }}^{2*}=\bar{\mu }(x^{2*})=[1,0]$. The line linking these two points is called the Utopia line (see Fig 5).

Next, we define ${\bar{N}}_{1}$ as the direction from ${\bar{\mu }}^{1*}$ to ${\bar{\mu }}^{2*}$, yielding ${\bar{N}}_{1}={\bar{\mu }}^{2*}-{\bar{\mu }}^{1*}=[1,-1]$. After that, we generate a predefined number of points ${\bar{X}}_{j}$ (*j* = 1, 2, …, *m*_1_, where *m*_1_ is the predefined number) along the direction of ${\bar{N}}_{1}$ distributed on the Utopia line by the following equation:

$${\bar{X}}_{j}=\alpha_{1j}{\bar{\mu }}^{1*}+\alpha_{2j}{\bar{\mu }}^{2*}$$ (27)

where 0 ≤ *α*_1*j*_,*α*_2*j*_ ≤ 1 and *α*_1*j*_ + *α*_2*j*_ = 1. Please note that for each *j* ∈ {1,2,…,*m*_1_}, *α*_1*j*_ is incremented by the step 1/(*m*_1_−1) while *α*_2*j*_ is decremented by the same step. Therefore, these points are evenly distributed on the Utopia line with a step of $\sqrt{2}/(m_{1}-1)$.

Using the set of evenly distributed points on the Utopia line, we can generate a corresponding set of Pareto points by solving a succession of optimization runs of the following single objective sub-problem:

*Single objective sub-problem* (for *j*th point)

- ***Objective function***

$$\min\;\bar{z}={\bar{\mu }}_{2}$$ (28)

- ***Subject to***

Formulae (18) ~ (25)

$${\bar{N}}_{1}{\left(\bar{\mu }-{\bar{X}}_{j}\right)}^{T}\leq 0$$ (29)

$$\bar{\mu }={\left[{\bar{\mu }}_{1}(x)\;{\bar{\mu }}_{2}(x)\right]}^{T}$$ (30)

Each optimization run corresponds to a point on the Utopia line. Specifically, for each generated point on the Utopia line, solve for the *j*th point.

Finally, after solving the *m*_1_ single objective sub-problems, we obtain *m*_1_ optimal solutions $x_{1}^{*},x_{2}^{*},\ldots ,x_{m_{1}}^{*}$. By substituting the optimal solutions into original objective functions *μ*_1_ and *μ*_2_, we get the set of Pareto points: $P_{1}=\left\{[\mu_{1}\;(x_{1}^{*})\;\mu_{2}\;(x_{1}^{*})],[\mu_{1}\;(x_{2}^{*})\;\mu_{2}\;(x_{2}^{*})],\ldots ,[\mu_{1}\;(x_{m_{1}}^{*})\;\mu_{2}\;(x_{m_{1}}^{*})]\right\}$.

### 4.2. Modified pareto filter algorithm

As indicated by Messac et al. [34], under certain circumstances, the normalized normal constraint method can generate non-Pareto solutions. Therefore, it is necessary to develop a Pareto filter algorithm to eliminate all dominated points from the given set of Pareto points (e.g. the set of P_1_); that is, to generate a set of global Pareto optimal points. Messac et al. [34] proposed a Pareto filter algorithm with a four-step process. However, according to our computational experiments, the algorithm fails to provide a set of Pareto points without repeated elements (Pareto points). That is to say, if the original set P_1_ contains repeated Pareto points, the algorithm cannot eliminate repeated copies. Moreover, according to the algorithm description (with both the pseudocode and the flow diagram), the original algorithm seems to occur a subscript out of range error when the subscript of the Pareto set (represented by i in [34]) is equal to the number of generated solutions (represented by m in [34]). This motivates us to enhance the algorithm with slight modifications. Table 1 presents the overall framework of our modified Pareto filter algorithm.

**Table 1:** Framework of the modified Pareto filter algorithm.

**Algorithm:** Modified Pareto Filter		
**Input:** Original set of Pareto points (*P*_1_)		
**Output:** Set of global Pareto optimal points without repeated elements (*P*_3_)		
**1:**	Flag ← zeros(*m*_1_)	// Flag is to flag repeated points
**2:**	*P*_2_ ← {}	// *P*_2_ is to store unrepeated points
**3:**	*P*_3_ ← {}	
**4:**	**for all***i* ∈ {1,2,…,*m*_1_} **do**	
**5:**	**if** Flag[*i*] = 0 **then**	
**6:**	**for all***j* ∈ {1,2,…,*m*_1_} **do**	
**7:**	**if***j* > *i***then**	
**8:**	**if***P*_1_[*j*] = *P*_1_[*i*] **then**	
**9:**	Flag[*j*] ← 1	
**10:**	**end if**	
**11:**	**end if**	
**12:**	**end for**	
**13:**	** end if**	
**14:**	**if** Flag[*i*] = 0 **then**	
**15:**	*P*_2_.add(*P*_1_[*i*])	// add *P*_1_[*i*] to set *P*_2_
**16:**	** end if**	
**17:**	**end for**	
**18:**	*m*_2_ ← |*P*_2_|	// *m*_2_ is the number of points in *P*_2_
**19:**	**for all***i* ∈ {1,2,…,*m*_2_} **do**	
**20:**	**for all***j* ∈ {1,2,…,*m*_1_} **do**	
**21:**	**if***j* ≠ *i***then**	
**22:**	**if***P*_2_[*i*] ≥ *P*_2_[*j*] **then**	
**23:**	**break**	
**24:**	**else**	
**25:**	**if***i* < *m*_2_**then**	
**26:**	**if***j* = *m*_2_**then**	
**27:**	*P*_3_.add(*P*_2_[*i*])	
**28:**	** end if**	
**29:**	**else**	
**30:**	**if***j* = *m*_2_−1 **then**	
**31:**	*P*_3_.add(*P*_2_[*i*])	
**32:**	** end if**	
**33:**	**end if**	
**34:**	**end if**	
**35:**	** end if**	
**36:**	** end for**	
**37:**	**end for**	

As described in Table 1, the overall algorithm framework consists of two basic modules: eliminating repeated points (Line 4 ~ 17) and eliminating dominated points (Line 19 ~ 37). Since the second-stage model is a combinational optimization problem, which means its solutions are discrete, it is possible to yield repeated points. This supposition is proved to be reasonable by our numerical experiments presented in Section 5. After eliminating all repeated points and dominated points (local Pareto optimal points), we can obtain the "real" Pareto frontier (global Pareto optimal points) from which decision makers can select a suitable configuration conveniently.

## 5. Computational experiments

In this section, in order to evaluate the two-stage model and the Pareto frontier generation algorithm, we carry out several computational experiments. We focus on two aspects: entire computational process for the two-stage model and a regression analysis as well as a sensitivity analysis on input parameters. The first-stage model is solved by Gurobi 6.5.2; and the second-stage model is solved by the normalized normal constraint method and each single objective sub-problem is also solved by Gurobi solver. All the computational experiments are coded in Python 2.7 and implemented within the interactive Python development environment of Spyder on a PC with Intel Core i5 CPU and 8 GB RAM. We start with the demonstration of the overall solution process.

### 5.1. Demonstration of the overall solution process

In order to demonstrate the overall solution process for the two-stage model, we are given the predefined shunting schedule presented in Fig 3. It is observed that there are a total of 23 shunting movements (shunting tasks) within the shunting schedule. The attributes for the shunting tasks, including start track, end track, start point, end point, start time and end time, are listed in Table 2.

**Table 2:** Shunting tasks deriving from Fig 3.

Task ID	Start Track	End Track	Start Point	End Point	Start Time ^a^	End Time ^a^
**1**	m2	s4	3	20	223	228
**2**	w1	m2	9	4	68	73
**3**	m3	s1	5	14	295	300
**4**	w2	m3	11	6	133	138
**5**	w1	m1	9	2	541	546
**6**	s2	w1	15	10	468	473
**7**	m2	s8	3	28	383	388
**8**	w1	m2	9	4	228	233
**9**	m4	s9	7	30	388	393
**10**	w2	m4	11	8	233	238
**11**	w1	s3	9	18	463	468
**12**	s3	m4	17	8	584	589
**13**	s3	w1	17	10	398	403
**14**	w1	m4	9	8	393	398
**15**	s7	w1	25	10	328	333
**16**	w2	m1	11	2	317	322
**17**	m3	s8	5	28	579	584
**18**	w1	m3	9	6	323	328
**19**	w2	m2	11	4	619	624
**20**	s6	w2	23	12	546	551
**21**	w1	m3	9	6	624	629
**22**	s5	w1	21	10	551	556
**23**	w2	m2	11	4	403	408

^a^ Unit: min.

The corresponding train depot used for our computational experiments is depicted in Fig 2. As shown in the figure, there are 15 depot tracks and 31 shunting driver involved points, including 30 getting on/off points and one shunting driver's lounge (i.e., the departure/arrival point for the shunting drivers). The walking distance between each two points is presented in S1 Table, and the walking speed of the shunting drivers is set as 90.0 m/min. In fact, the illustrated depot is a simplified version of Shanghai South Depot, China. Therefore, all the computational results can serve as a benchmark and a decision support for real-life problems.

**Fig 6:**
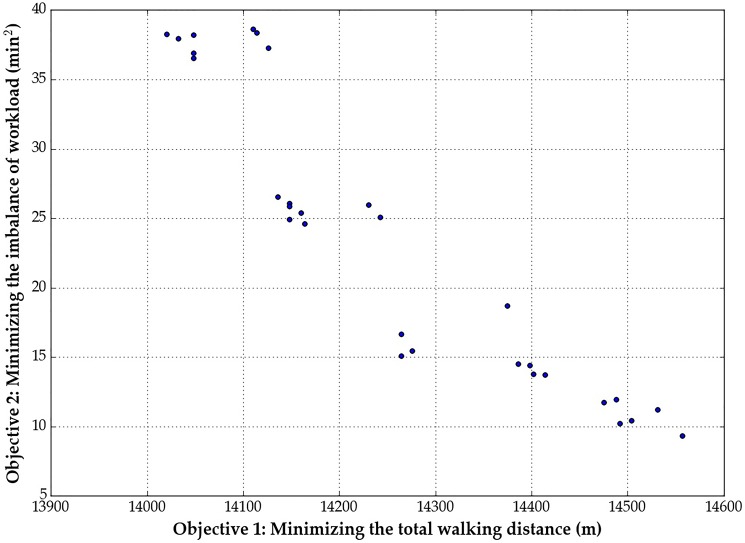
Pareto points without the Pareto filter.

**Fig 7:**
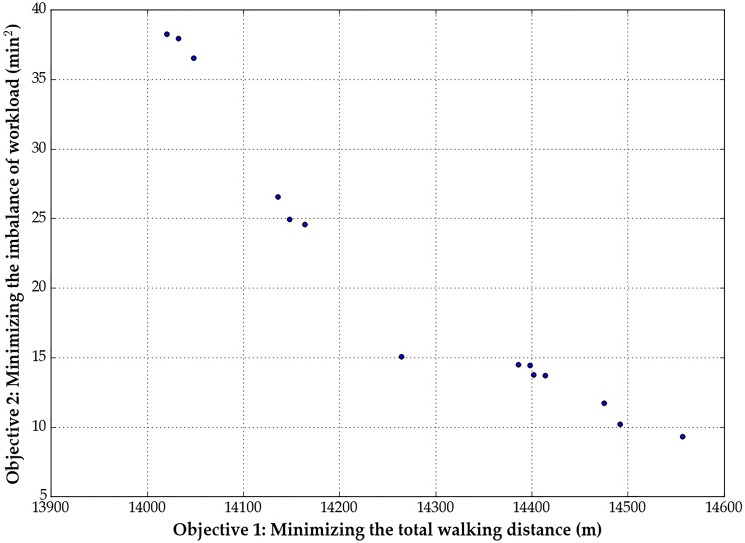
Pareto points with the Pareto filter.

Given the input data, we can solve the first model using the optimization solver Gurobi. The solution time is just a matter of seconds. The optimal objective value of the first-stage model is 3, which means the optimal workforce size of shunting drivers for the 23-task instance is 3. With the optimal workforce size, we can proceed to solve the second-stage BOP. We are interested in obtaining the Pareto frontier for the BOP. Solving the above problem instance with a normalized increment of 0.01 yields 101 Pareto solutions. Fig 6 shows the generated Pareto solutions by the normalized normal constraint method. Among these solutions, there are a total of 71 repeated copies, which corresponds with our supposition that it is possible to generate repeated points since the problem is a combinational optimization problem. By performing the modified Pareto filter algorithm, we can further identify 16 non-Pareto optimal solutions. As a result, there are only 14 global Pareto points left after the entire solution process. The global Pareto points are shown in Fig 7.

As can be seen in Fig 7, the range for the first objective function's value is between 14,000 and 14,600 (m) while the range for the second objective is between 5 and 40 (min^2^). When the total walking distance for all shunting drivers decreases, the imbalance of workload increases, which means the two objectives are conflicting. If we consider the optimal walking distance (overall costs) as the "system optimal (global optimal)" objective and regard the optimal workload balance (fairness) as the "user equilibrium (local optimal)" objective, this figure could reveal why "local optimal" does not always mean "global optimal", and vice versa. For decision makers, they should comprehensively consider the fairness (Objective 2) and overall costs (Objective 1), i.e., making a suitable tradeoff between these two goals. Once the decision makers have made a suitable choice, they can select a configuration from the Pareto frontier directly. For example, if the decision makers want the imbalance level be lower than 15, they should select the seven Pareto points distributed at the lower right corner of Fig 7.

To highlight the proposed approach on both aspects of solution quality and speed, in Table 3 we compare the computational results with those from the manual method based on above case. In terms of the solution quality, we focus on the objective values (including driver size, total walking distance and workload imbalance). While with respect to the solution speed, we pay our attention to the total time it takes to design a shunting driver assignment plan.

**Table 3:** Comparisons between the manual method and proposed approach.

Criterion	Manual Method	Proposed Approach	Gap
**Driver Size**	4	3	1
**Walking Distance (m)**	14820.5	Pareto solutions (14020.5 ~ 14557.5)	-
**Workload Imbalance (min**^**2**^**)**	267.0632	Pareto solutions (9.3117 ~ 38.2193)	-
**Computational Time**	2 ~ 3 h	4.05 sec^a^	-

^a^ The computational time of the first-stage model is 0.03 sec; and the second-stage model is 4.02 sec.

As can be seen from Table 3, the optimal shunting driver size from the manual method is four, which means the manual plan requires one more driver to complete all the shunting tasks. Since labor costs represent a large share of rail operators' operating costs [36], reducing the workforce size is beneficial for rail operators to pursuit operating cost savings. Regarding the total walking distance of all shunting drivers, the two methods show little difference. However, for the workload imbalance criterion, the proposed approach significantly improves the balance level by approximately ten times compared with the manual method. Moreover, the proposed approach is able to provide the Pareto solution pool while the manual method just yields a single plan. Another advantage of the proposed approach embodies in the solution speed. According to our field investigations at Shanghai Depot, it generally takes the dispatcher two to three hours to design a shunting driver assignment plan. While it just requires four seconds through automatic computing using the proposed approach. In practice, our method could provide a useful and efficient decision support tool for dispatchers whenever designing a new plan or rescheduling an existing plan.

### 5.2. Discussions

#### 5.2.1. Regression analysis for the first-stage model

It is interesting to investigate how the input data (parameters) impact the optimization results (optimal workforce size of shunting drivers). To this end, we estimate a statistical regression model that can produce significant predictions of the workforce size based on a small number of problem characteristics, which are listed in Table 4. We suspect the listed characteristics to have the gravest impact on the optimal workforce size.

**Table 4:** Problem characteristics.

No.	Characteristic Description	Abbreviation
**1**	Number of shunting tasks	NST
**2**	Average walking distance between any two shunting tasks	AWD
**3**	Number of infeasible shunting task connections	NIC

The total number of shunting tasks is an obvious choice, since more shunting tasks always require more shunting drivers. The average walking distance between any two shunting tasks is also rather an evident selection, because for a given walking speed and a predefined planning horizon, the longer walking distance between two shunting tasks implies less number of shunting tasks a shunting driver can handle, which means more shunting drivers are needed. Finally, we pay our attention to the total number of infeasible shunting task connections. An infeasible shunting task connection refers to that the walking time for a shunting driver is longer than the connection time defined by the shunting schedule between a pair of shunting tasks. Therefore, larger number of infeasible shunting task connections represent less flexibility for shunting drivers. As a result, extra shunting driver(s) are necessary to avoid this type of infeasibility. Consider an extreme scenario where any two shunting tasks are infeasible connections, then the optimal driver size will be equal to the total number of shunting tasks.

We generate 25 shunting task plans and solve them with the proposed method. Note that for simplification reasons and without loss of generality, we still focus on the pre-described depot layout (see Fig 2). We then estimate a linear regression model based on the three characteristics for the dataset using Microsoft Excel. Table 5 presents the estimated parameters calculated from the dataset. The regression model features a coefficient of determination *R*^2^ of 0.905, which indicates that 90.50% variability is explained by the model. Moreover, all the *p*-values of the selected three problem characteristics (coefficients) are much less than 0.05, which means the characteristics have a strong linear relationship with the optimal workforce size of shunting drivers, i.e. indicating high significance.

**Table 5:** Coefficients and std. errors for the regression model^a^.

Parameter	Denotation	Coefficient	Std. Error	*p*-value
**Intercept**	*β*_0_	-0.3200	0.2249	0.1693
**NST**	*β*_1_	1.1690	0.1222	<0.0001
**AWD**	*β*_2_	-0.0041	0.0005	<0.0001
**NIC**	*β*_2_	-0.0086	0.0023	0.0013

^a^ The confidence interval is set as 95%.

As can be seen from Table 5, the number of shunting tasks (NST) is the most important factor that impacts the optimization results. Therefore, we are particularly interested in how the number of shunting tasks impacts the Pareto frontier for the second-stage model. We will conduct the interesting sensitivity analysis in the following sub-section.

#### 5.2.2. sensitivity analysis for the second-stage model

**Fig 8:**
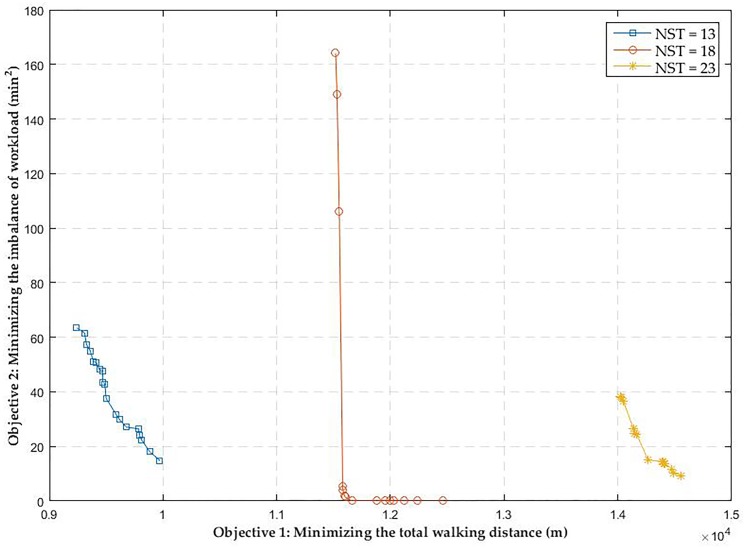
Sensitivity analysis on the number of shunting tasks.

In this section, we would like to investigate how the Pareto solutions of the second-stage BOP change with the number of shunting tasks. To quantify the impact, we select three data samples (shunting task plans) from the above mentioned dataset, for which the optimal solutions of the first-stage model are all equal to three (shunting drivers), but the number of shunting tasks for the three plans are 13, 18 and 23, respectively. The Pareto solutions for the selected problem instances are shown in Fig 8. Please note that in the figure, the lines linking two adjacent Pareto points do not represent the Pareto frontiers, they are just for visualization purposes.

Fig 8 clearly shows that the Pareto optimal curves move from left to right as the number of shunting tasks (NST) increases from 13 to 23. It implies that, at the same workload balance level, a larger NST leads to a longer total walking distance. The reason is quite simple. Since the number of shunting driver is a constant, a larger total NST means that a shunting driver is assigned more shunting tasks. Consequently, the total walking distance of all shunting drivers increases.

## 6. Conclusions

In this paper, we propose a two-stage approach to the EMU depot shunting driver assignment problem, aiming to link two research highlights in the transportation area: the crew sizing problem and the crew scheduling problem. The first-stage model is to minimize the workforce size of shunting drivers, which is a primary goal for the decision makers. With the optimal driver size, the second-stage model comprehensively consider the overall costs (total walking distance) as well as fairness (workload balance), which is modeled as a bi-objective problem.

We apply the normalized normal constraint method to solve the second-stage BOP. The method consists of two modules: Pareto point generation and non-Pareto solution filter. Motivated by the disadvantage of original Pareto filter algorithm, we develop an enhanced Pareto filter algorithm with slight modifications.

Furthermore, to evaluate the proposed model and algorithm, we carry out several computational experiments. Discussions with respect to computational results provide some interesting insights, including: (1) a linear regression model with a high coefficient of determination for the first-stage sub-problem is estimated, the regression parameters can be useful for decision makers to roughly determine a workforce size of shunting drivers while just using three easily obtainable problem characteristics; (2) a sensitivity analysis for the second-stage model is conducted and we observe that the Pareto frontier moves from left to right as the total number of shunting tasks increases.

Our future research direction is to simultaneously consider the depot shunting scheduling problem and the shunting driver assignment problem. We would like to use a bi-level programming framework or other advanced modeling techniques to address the integrated optimization problem.

## Supporting information

S1 Table Walking distance between each two points (unit: m). (XLSX)
